# One-Year Consumption of a Mediterranean-Like Dietary Pattern With Vitamin D3 Supplements Induced Small Scale but Extensive Changes of Immune Cell Phenotype, Co-receptor Expression and Innate Immune Responses in Healthy Elderly Subjects: Results From the United Kingdom Arm of the NU-AGE Trial

**DOI:** 10.3389/fphys.2018.00997

**Published:** 2018-07-26

**Authors:** Monica Maijo, Kamal Ivory, Sarah J. Clements, Jack R. Dainty, Amy Jennings, Rachel Gillings, Susan Fairweather-Tait, Massimo Gulisano, Aurelia Santoro, Claudio Franceschi, Simon R. Carding, Claudio Nicoletti

**Affiliations:** ^1^Gut Health Programme, Institute Strategic Programme, Quadram Institute Bioscience, Norwich, United Kingdom; ^2^Norwich Medical School, University of East Anglia, Norwich, United Kingdom; ^3^Department of Experimental and Clinical Medicine, University of Florence, Florence, Italy; ^4^Department of Experimental, Diagnostic and Specialty Medicine, University of Bologna, Bologna, Italy; ^5^C.I.G. Interdepartmental Centre “L. Galvani”, University of Bologna, Bologna, Italy

**Keywords:** aging, dietary intervention, nutrition, inflammation, elderly, NU-AGE

## Abstract

Amongst the major features of aging are chronic low grade inflammation and a decline in immune function. The Mediterranean diet (MedDiet) is considered to be a valuable tool to improve health status, and although beneficial effects have been reported, to date, immunological outcomes have not been extensively studied. We aimed to test the hypothesis that 1 year of a tailored intervention based on the MedDiet with vitamin D (10 μg/day) would improve innate immune responses in healthy elderly subjects (65–79 years) from the English cohort (272 subjects recruited) of the NU-AGE randomized, controlled study (clinicaltrials.gov, NCT01754012). Of the 272 subjects forming the United Kingdom cohort a subgroup of 122 subjects (61 in the intervention group and 61 in the control group) was used to evaluate *ex vivo* innate immune response, phenotype of circulating immune cells, and levels of pro- and anti-inflammatory markers. Odds Ratio (OR) was calculated for all the parameters analyzed. After adjustment by gender, MedDiet-females with a BMI < 31 kg/m^2^ had a significant upregulation of circulating CD40^+^CD86^+^ cells (OR 3.44, 95% CI 1.01–11.75, *P* = 0.0437). Furthermore, in all MedDiet subjects, regardless of gender, we observed a MedDiet-dependent changes, although not statistically significant of immune-critical parameters including T cell degranulation, cytokine production and co-receptor expression. Overall, our study showed that adherence to an individually tailored Mediterranean-like dietary pattern with a daily low dose of vitamin D3 supplements for 1 year modified a large variety of parameters of immune function in healthy, elderly subjects. We interpreted these data as showing that the MedDiet in later life could improve aspects of innate immunity and thus it could aid the design of strategies to counteract age-associated disturbances.

**Clinical Trial Registration:** clinicaltrials.gov, NCT01754012.

## Introduction

Aging is an irreversible process that is associated with a significant decline of the immune system that leads to increased susceptibility to infection, reduced efficiency of vaccination protocols and significantly higher incidence of autoimmune disorders and cancer (Castle, 2000; Dewan et al., 2012). Chronological age may also result in vascular aging with deterioration in arterial function (Jani and Rajkumar, 2006) associated with cardiovascular diseases (CVD) that are the most common world-wide cause of morbidity and mortality in persons aged over 65 years, particularly those with diabetes mellitus. The negative impact on vascular function compromises the ability to resist oxidative stress and inflammation. Aging is characterized by a chronic, low grade inflammatory status termed “inflammaging” (Franceschi et al., 2000, 2007; Monti et al., 2017), which plays a critical role in the pathogenesis of major age-related chronic diseases and in important geriatric conditions (De Martinis et al., 2006; Pedersen, 2009; Lencel and Magne, 2011; Franceschi and Campisi, 2014) and in so doing contributes to both a poor quality of life and elderly mortality (Cevenini et al., 2013; Varadhan et al., 2014). Increased production of inflammatory mediators and the simultaneous down-regulation of anti-inflammatory ones takes place in a variety of tissues, organs and systems including adipose tissue, muscles, brain, and contribute to the onset and progression of such a systemic inflammatory state (Cevenini et al., 2010, 2013).

Immunosenescence is a hallmark of the aging organism and both the innate and adaptive arms of the mucosal and systemic immune systems are affected (Maijó et al., 2014; Man et al., 2015), with alterations in constituent cells linked to the onset of chronic diseases and it is generally accepted that immunosenescence is accelerated in those subjects who present inflammaging. The design of interventional strategies to delay, halt or even possibly reverse immunosenescence requires a detailed understanding of the aging process at cellular level.

Although the individual genetic make-up that dictates immune responses cannot be modified, other factors contributing to aging such as lifestyle and diet (Pae et al., 2012) could be changed in the attempt to halt/reverse the progression of the detrimental effects of aging on immune effectiveness (Franceschi et al., 2017). To this end, the adoption of nutritional regimen to improve immune function in the elderly is a potentially important approach. Indeed, a diet rich in fruit, vegetables, legumes, unrefined cereals and olive oil and accompanied by low intake of meat, dairy products and alcohol consumption typical of the Mediterranean is effective in decreasing the incidence of cardiovascular and other chronic diseases and increased longevity (Mena et al., 2009; Vasto et al., 2012; Casas et al., 2014, 2018; Martinez-Gonzalez et al., 2015; Santangelo et al., 2018).

The recognition of nutrition as a possible determinant of successful aging was acknowledged by the European Union by funding a dietary intervention study, the NU-AGE project ^1^. A multidisciplinary European consortium developed a tailored dietary strategy to address the needs of the elderly (65–79 years) for promoting healthy aging in Europe. The dietary strategy included the daily supplementation of a low dose of vitamin D (10 μg or 400 IU/day) to minimize differences between study sites part of the whole NuAge project across different areas of Europe that differed in the diet and exposure to sunlight, two very critical factors for determining the vitamin D status. The aim of the study was to examine the effect of consuming a Mediterranean-like dietary pattern (NU-AGE diet) with vitamin D3 for 1 year on a large variety of parameters of immunity that included immune cell phenotype, co-receptor expression and innate immune responses.

## Materials and Methods

### Participants

This study was a part of the larger multi-national NU-AGE study, a parallel-group, multi-centric, randomized, controlled clinical trial of 1-year duration (clinicaltrials.gov, NCT01754012) aimed at assessing the effects of the MedDiet in healthy aging [European seven framework project (FP7); Grant Agreement no. 266486^2^]. Full details of the study protocol have been described elsewhere (Santoro et al., 2014). It was not possible to blind the study to the participants or scientists responsible for managing the dietary intervention, but all the sample analysis was blinded. All subjects provided written informed consent prior to their inclusion in the study. This study reports data from 272 subjects recruited at baseline in the NU-AGE English cohort (134 control, 138 MedDiet). One arm of the study aimed to study the effects of dietary intervention on a variety of parameters of the innate immune system. To this end, 122/272 subjects of the United Kingdom cohort were utilized to study immune responses, (61 control, 61 MedDiet). After 1 year, 115/122 subjects, among those studied for immune responses, completed the intervention. Participants were independently living in their own home. Specific inclusion criteria were as follows: men or women aged 65–79 years; with no chronic disease, such as cancer, organ failures for the last 2 years; responsible for own meal choices and preparation; willing to make changes to their habitual diet for 1 year. Exclusion criteria were: current disease (e.g., cancer or dementia); organ failure or organ failure requiring a special diet; the following chronic diseases: severe heart disease, kidney disease, respiratory insufficiency, liver cirrhosis; diabetes Mellitus type 1. Also, exclusion criteria included long-term use of corticosteroids, use of non-steroidal anti-inflammatory drugs, use of antibiotics within the previous 2 months, change in habitual medication use (e.g., statins, thyroxin) within 3 months prior the beginning of the study, participation in another current study involving either dietary interventions or sampling/donation of blood, malnutrition (BMI lower than 18.5 kg/m^2^); body weight loss of more than 10% within 6 months. Participants were assigned randomly (1:1) to the control or intervention groups, after stratification by sex, age (65–72 or >72–79 years), frailty status (pre-frail or non-frail), and body mass index (<25 or ≥25 kg/m^2^). Randomization was done by computer-generated allocation and ran on Python interpreter (version 2.6 or 2.7). The majority of the subjects (81%) were non-frail; the remainder were pre-frail (19%).

### Dietary Intervention

The NU-AGE dietary intervention has been previously described (Berendsen et al., 2014). In brief, all participants (control and intervention groups) completed a 7-day food record diary within 2 weeks after the baseline assessment. This was followed by an interview with a specialized team member to review the records. The consumed food recorded in the food diary was coded according to standardized coding procedures (Berendsen et al., 2014). The control group, following a habitual diet, received only a leaflet with national dietary guidelines. The intervention group was given individually tailored Mediterranean diet advice plus a daily vitamin D_3_ supplement of 10 μg. The combination of the Mediterranean dietary pattern and vitamin D is henceforth referred to solely as MedDiet. The nutritional intervention was from the Food Based Dietary Guidelines for older adults (Berendsen et al., 2014) conducted by a dietician/nutritionist using individual education using a Motivational Intervention technique. Participants were advised to consume on average 15–20% of their daily energy from protein, 50–60% from carbohydrate, 25–30% from fat (<10% from saturated fat, <1% from trans fatty acids, <12% from polyunsaturated fatty acids, and 8–28% from monounsaturated fatty acids). In addition, subjects were recommended to consume 30–40 g/day of fiber, <10–20 g/day of preferably red wine (1 serving/day for women and 2 for men), 2 g/day sodium, 1.3 g/day potassium, 10 mg/day iron, 15 μg/day of vitamin D_3_, 400 μg/day of folate, and 5 μg/day of vitamin B12. The MedDiet cohort (*n* = 61) received individual, tailored dietary advice (summary in **Table 1**) based on dietary reference values in the United Kingdom ^3^ and recommendations from bodies including the modified My Pyramid for Older Adults (Peregrin, 2008; Shelnutt et al., 2009), European Commission (Commission of the European Communities and Nutrient and Energy Intakes for European Community, 1993), and Institute of Medicine (Institute of Medicine of the National Academies and Dietary Reference Intakes, 2006). Subjects in the control group (*n* = 61) were given a standard healthy-living advice leaflet from the British Dietetic Association and asked to maintain their normal dietary habits over the course of the year.

**Table 1:** Quantitative dietary guidelines given to study participants.

Food group	Quantity required in dietary intervention
Whole grains	6 servings per day; 1 serving = 25 g bread, 50 g breakfast cereal
Fruits	2 servings per day; 1 serving = 1 apple, 1 banana, 8 small plums
Vegetables and legumes	330 g per day, once per week 200 g legumes
Dairy and cheese	500 ml dairy per day (of which 30 g cheese)
Fish and other seafood	2 times per week; 1 portion = 125 g
Meat and poultry	4 times per week; 1 portion = 125 g
Nuts	2 times per week; 20 g portion
Potatoes, pasta and rice	150 g per day; 80 g (raw weight) whole grain rice or pasta at least twice a week
Eggs	2–4 times per week
Oil or fat	20 g oil per day, 30 g margarine per day; maximum of 50 g fat per day. Should be olive oil and low fat margarine rich in MUFA and PUFA
Alcohol	Maximum of 1–2 glasses per day for men, and 1 glass per day for women. Preferably red wine, if not abstain
Fluid	1.5 liter per day, including milk
Salt	Reduce added salt, and intake of ready meals (soups, gravy, and sauce)
Sugar	Limit consumption of sugar and sweetened drinks (replace with fruit or yogurt, no/reduce sugar in tea or coffee).


### Isolation of Mononuclear Cells

Blood collected in vacutainers (Becton Dickinson, Oxford, United Kingdom) containing ethylenediaminetetraacetic acid (EDTA) was centrifuged at 2200 rpm to separate plasma from the pelleted cells. Plasma was collected and stored at -80°C for future analyses. Cell pellets were diluted 1:3 with Hank’s Balanced Salt Solution (HBSS; Sigma-Aldrich, Dorset, United Kingdom) and peripheral blood mononuclear cell (PBMCs) were separated using Leucosep tubes according to the manufacturer instructions (Greiner Bio-one). PBMCs were collected from the interface, washed and cell counts were adjusted to 4 × 10^6^ viable cells/mL with ‘complete’ DMEM/F12 (Life Technologies Ltd., Paisley, United Kingdom) supplemented with 100 U/mL penicillin, 100 μg/mL streptomycin, and 50 mL of FBS (Sigma, United Kingdom).

### Co-receptor Expression Induced by Toll-Like Receptor (TLR) Agonists

Peripheral blood mononuclear cells at 1 × 10^6^ viable cells/mL (final concentration) in complete DMEM/F12 were cultured with 50 μg/mL Poly A:U, 2.5 μg/mL Imiquimod-R837, 25 μg/mL ODN-M362, 5 μg/mL LPS-ED and 50 ng/mL Pam2CSK4 (Invivogen, San Diego, CA, United States) or without for 22 h in 24-well flat-bottom plates at 37°C in an atmosphere of 5% CO_2_ in air. Cell-free supernatants were collected and stored at -80°C until analysis. Freshly collected cells were washed and stained for expression of costimulatory molecules by incubation for 20 min at 20°C with fluorochrome-labeled monoclonal antibodies (mAbs): anti-CD154 Brilliant Violet (BV) 421 (Biolegend, San Diego, CA, United States), anti-CD80 Alexa Fluor (AF488) (Biolegend), anti-CD86 Allophycocyanin (APC) (Biolegend), anti-HLA-Dr AF700 (Biolegend), anti-CD152 Phycoerythrin (PE) (Biolegend), anti-CD40 Phycoerythrin-Cyanine 7 (PE/Cy7) (Biolegend), and anti-CD1d APC (Biolegend). Negative control cells were incubated with the corresponding isotype-matched negative control mAbs conjugated with BV421, AF488, APC, AF700, PE/Cy7, and PE (Biolegend). Cells were washed, then fixed with Formalin 1X, before analysis by flow cytometry (EC800 Sony Biotechnology Inc., Surrey, United Kingdom). Analysis was performed using FlowJo software (FlowJo, Oregon, United States).

### *In Vitro* Responses to TLR Agonists

Culture supernatants were collected following culture with TLR agonists as described above. Quantitative determination of IFN-α, IFN-β, IFN-γ, IL-12p40, IL-12p70 present in the culture supernatants was performed by ProcartaPlex^TM^ Immunoassay (eBioscience, United Kingdom) according to the manufacturer’s instructions and analysis using Luminex 200 instrumentation (Luminex Corporation, Netherlands). The immunoassay kits were specific for human cytokines. Assay sensitivities were 0.6 pg/mL for IFN-α, 8.54 pg/mL for IFN-β, 8.89 pg/mL for IFN-γ, 1.81 pg/mL for IL-12p40, and 6.30 pg/mL IL-12p70. The soluble expression of SOCS3, IL-12Rβ1, and IL-12Rβ2 in the supernatants was performed using enzyme-linked immunosorbent assay (ELISA) kits (Antibodies-online, Atlanta, GA, United States) according to the manufacturer’s instructions. The assay sensitivities were 15.6 pg/mL for SOCS3, 0.156 ng/mL for IL-12Rβ1, and 0.156 ng/mL IL-12Rβ2 and ELISA plates were read in a microplate reader at 450 nm (Benchmark Plus Bio-Rad, Hercules, CA, United States).

### T Cell Proliferation

Peripheral blood mononuclear cells at 2 × 10^6^ viable cells/mL (final concentration) in complete DMEM/F12 were cultured with 5 μg/mL of Staphylococcal enterotoxin B (SEB; Sigma-Aldrich, United Kingdom) for 6 days in 24-well flat-bottom plates at 37°C and 5% CO_2_. Cells were stained with anti-CD3 BV421 (Biolegend), and anti-CD8 AF488 (Biolegend) for 20 min at 20°C. T cell proliferation was measured using the Coulter DNA Prep Reagent Kit (Beckman Coulter Ltd., High Wycombe, United Kingdom) according to the manufacturer’s instructions. Data were acquired by Flow cytometry (EC800 Sony). Analysis was performed using FlowJo software (FlowJo, United States).

### Dendritic Cell Subtypes Enumeration/Ratio

DCs were enumerated in heparinized blood using a Blood Dendritic Cell Enumeration Kit (Miltenyi Biotec Ltd., Surrey, United Kingdom) according to the manufacturer’s instructions. The anti-BDCA cocktail contains: anti CD303 (BDCA-2) Fluorescein (FITC), anti-CD1c (BDCA-1) PE, anti-CD14 Phycoerythrin-Cyanine 5 (PE-Cy5), and anti-CD19 PE-Cy5 for the determination of plasmacytoid (pDC) and myeloid (mDC) DC. Antibodies to CD14 and CD19 antigens were used for exclusion of monocytes and B cells, respectively. To enable quantification in terms of absolute counts/μL blood, Flow count fluorospheres (Beckman Coulter) were added to each sample prior to flow cytometric (EC800 Sony) data acquisition. Analysis was performed using FlowJo software (FlowJo, United States). The ratio of mDC to pDC were calculated by applying the following formula DC = number of DC/number count beads × concentration).

### CD8 T Cell Degranulation

Peripheral blood mononuclear cells at 1 × 10^6^ viable cells/ml (final concentration) in complete DMEM/F12 were placed into each of two sterile tubes labeled ‘test’ or ‘control.’ Cells in the ‘test’ tube were incubated with anti-CD107a AF647 (Biolegend) for 15 min, before addition of 1.1 μg/mL SEB (Sigma-Aldrich, United Kingdom) as stimulant and 5 μL ethanol. The ‘control’ sample was not stimulated but had 5 μL (1 μM) colchicine (Sigma, Missouri, United States) in ethanol added to minimize spontaneous degranulation. In both tubes the final volume was adjusted 1 mL with tissue media culture. Tubes were incubated for 5 h at 37°C and 5% CO_2_, then washed and stained with anti-CD3 AF488 (Biolegend) and anti-CD8 PE (Biolegend). After further washing and fixation with 1% formalin, data were acquired by flow cytometry (EC800 Sony) and analyzed using FlowJo software.

### Granulocyte Oxidative Burst Activity

The quantitative determination of leukocytes oxidative burst was performed in whole blood using the Phagoburst^TM^ kit (Glycotope Biotechnology, Heidelberg, Germany) according to the manufacturer’s instructions. Burst activity in leukocytes was evaluated after stimulation with *N*-formyl-methionineleucine-phenylalanine (fMLP), a moderate stimulus. The generation of ROS was measured by assessing the oxidative conversion of the fluorogenic substrate dihydorohomdamine-123 into rhodamine. Data were acquired by flow cytometry (EC800 Sony) and analyzed using FlowJo software.

### Plasma Cytokines

Plasma samples stored at -80°C were thawed for the quantitative determination of IL-2, IL-9, IL-22, IL-23 by ProcartaPlex^TM^ Immunoassay (eBioscience, Hatfield, United Kingdom) according to the manufacturer’s instructions. Analysis was performed using Luminex 200 instrumentation (Luminex Corporation, Netherlands). Assay sensitivities were 4.44 pg/ml for IL-2, 11 pg/ml for IL-9, 33 pg/ml for IL-22, 19 pg/ml for IL-23.

### T Cell Phenotyping

Peripheral blood mononuclear cells at 1 × 10^6^ viable cells/mL (final concentration) in complete DMEM/F12 were incubated with anti-CD3 AF700 (Biolegend), anti-CD28 Pacific blue (PB) (Biolegend), anti-CD161 PE-Cy7 (Biolegend), anti-CD158d PE (Biolegend), and anti-TCR Vα24-Jα18 FITC (Biolegend) or the corresponding isotype controls. All antibodies were purchased from Biolegend (United Kingdom). After 20 min at 20°C cells were washed, and fixed with 1% formalin. Data were acquired by flow cytometry (EC800 Sony) and analyzed using FlowJo software.

### Statistical Analysis

Primary endpoints of the NU-AGE study were changes in immune responses; and a variety of secondary endpoints (ClinicalTrials.gov, NCT01754012). This work focuses on the effects of MedDiet on parameters of immune function. Samples from all volunteers were run in triplicate for each assay. Associations were investigated based on an adjusted Mantel–Haenszel odds ratio (OR), for a post-study change in the intervention group compared to the controls, some analysis were stratified by gender. Associations were evaluated using a Mantel–Haenszel chi-squared test. Confidence intervals were calculated using the method of Robins–Breslow–Greenland. Sex interactions were evaluated using Tarone’s test of homogeneity. Odds ratios were calculated such that a value less than 1 corresponds to a reduction in the intervention arm compared to the control arm. *P*-values were considered significant at less than 0.05.

## Results

### Participants

**FIGURE 1:**
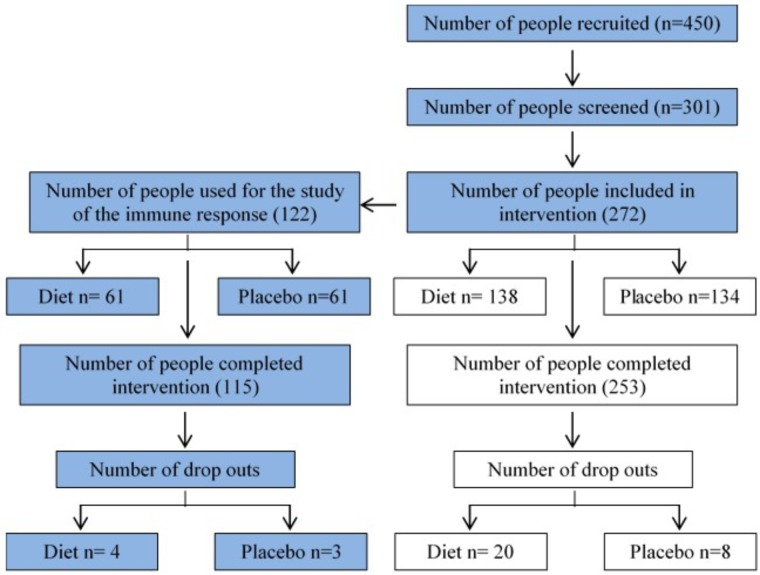
Flow chart summarizing recruitment and retention of study participants. 272 volunteers were recruited at baseline in the NU-AGE English (Norwich) cohort (134 control, 138 MedDiet) for the dietary intervention. This study (blue boxes) focuses on the effects of dietary intervention on a variety of parameters of the immune responses.

**Figure 1** shows the recruitment and retention of the volunteers. Quantitative dietary guidelines given to study participants and physical features of the cohort, such as age, body mass index (BMI) at baseline and at the end of the study, gender and percentage of pre-frail individuals of the cohort studied are detailed in **Tables 1, 2**, respectively.

**Table 2:** Physical features of the study participants.

Characteristics	Control *n* = 61	MedDiet *n* = 61	All subjects *n* = 122
Age (years)	70.63 ± 3.77	69.97 ± 4.20	70.27 ± 4.02
Body mass index (kg/m^2^) baseline	26.9 ± 3.3	26.4 ± 4.5	26.7 ± 4.0
Body mass index (kg/m^2^) final	26.7 ± 3.5	26.2 ± 4.4	26.4 ± 4.1
Women %	63.0	58.2	60.4
Pre-frail %	18.5	19.5	19


*Physical features of the participants were recorded at baseline and at the end of the 1-year dietary intervention period.*

### TLR Agonist Induced Co-receptor Expression and Cytokine Secretion

**FIGURE 2:**
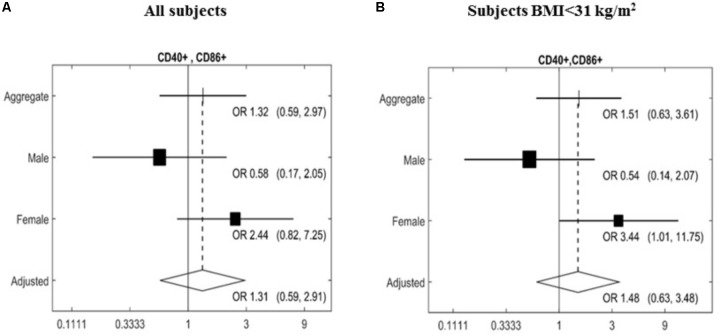
Effects of MedDiet on CD40^+^CD86^+^ expression following *in vitro* challenge with TLR agonists. PBMC were cultured with a cocktail of TLRs agonists (PolyA:U, Imiquimod, ODN-M362, LPS, Pam2CSK4) for 22 h. Forest plot of the study of the CD40^+^CD86^+^ lymphocytes comparing the association between Mediterranean diet and normal diet after 1 year of intervention with the random effect model. Associations were investigated based on an adjusted Mantel–Haenszel odds ratio (OR), stratified by gender **(A)** or BMI > 31 kg/m^2^ **(B)**. OR and 95%Cl were calculated. Cl, confidence interval.

The expression of a large panel of costimulatory molecules comprising CD154, CD80, CD86, HLA-Dr, CD40, CD152, and CD1d after *in vitro* challenge with TLR agonists was studied in lymphocyte and monocyte/DC populations. Expression of selected co-receptors monitored after stimulation is expressed as percentage (%) upregulation compared to expression on unstimulated resting cells. A gender-specific effect of the MedDiet was observed within the lymphocyte population with a significant upregulation of CD40^+^CD86^+^ cells (OR 3.44, 95% CI 1.01–11.75, *P* = 0.0437) observed in female study participants. Interestingly, this was restricted to individuals with BMI < 31 kg/m^2^ (**Figure 2**).

From all the other markers studied (CD154, CD80, CD86, HLA-Dr, CD40, CD152, and CD1d) no significant changes were found in the monocyte/DC populations (**Table 3**).

**Table 3:** Effects of MedDiet on the expression of co-stimulatory molecules.

Phenotype	OR (95%Cl)	*P-*value
CD154	0.84 (0.36–1.99)	0.6953
CD80	1.10 (0.50–2.41)	0.8053
CD86	1.07 (0.36–3.16)	0.9005
HLA-Dr	0.43 (0.18–1.06)	0.0655
CD40	0.92 (0.41–2.06)	0.8404
CD152	1.04 (0.45–2.37)	0.9313
CD1d	1.18 (0.52–2.69)	0.6859


*Peripheral blood mononuclear cell were cultured with a cocktail of TLRs agonists (PolyA:U, Imiquimod, ODN-M362, LPS, Pam2CSK4) for 22 h. Expression of the co-stimulatory molecules was monitored by flow cytometry Odd ratio (OR) and 95%Cl and p-values are shown.*

**FIGURE 3:**
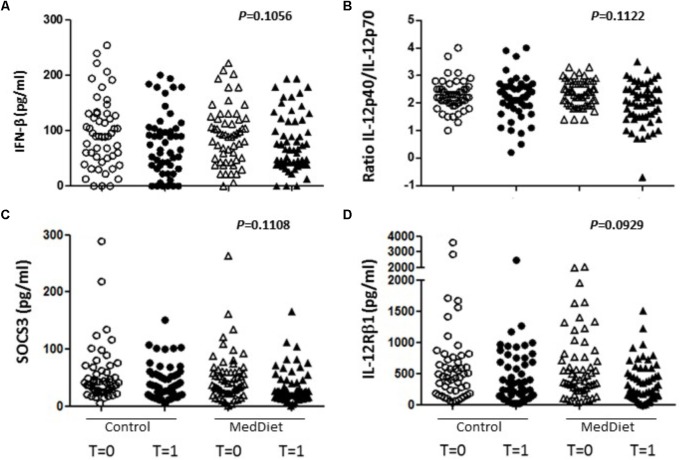
Effects of the MedDiet on cytokine expression following *in vitro* challenge with TLR agonists-all individual values. PBMC were cultured with a cocktail of TLRs agonists (PolyA:U, Imiquimod, ODN-M362, LPS, Pam2CSK4) for 22 h. Levels of cytokines were determined in culture supernatants. Representation of the individual values for IFN-β **(A)**, IL-12p40/70 ratio **(B)**, SOCS3 **(C)** and IL-12Rβ1 **(D)** concentration in all the volunteers at baseline and after 1 year of intervention. Results showed a tendency toward increased production of IFN-β (OR 1.98, 95%CI 0.86–4.53, *P* = 0.1056), and decreased production of IL-12Rβ1 (OR 0.50, 95%CI 0.22–1.13, *P* = 0.0929), SOCS3 (OR 0.50, 95%CI 0.21–1.18, *P* = 0.1108) and the ratio between IL-12p40/IL-12p70 (OR 0.54, 95%CI 0.26–1.16, *P* = 0.1122. OR = odds ratio), CI, confidence intervals.

**FIGURE 4:**
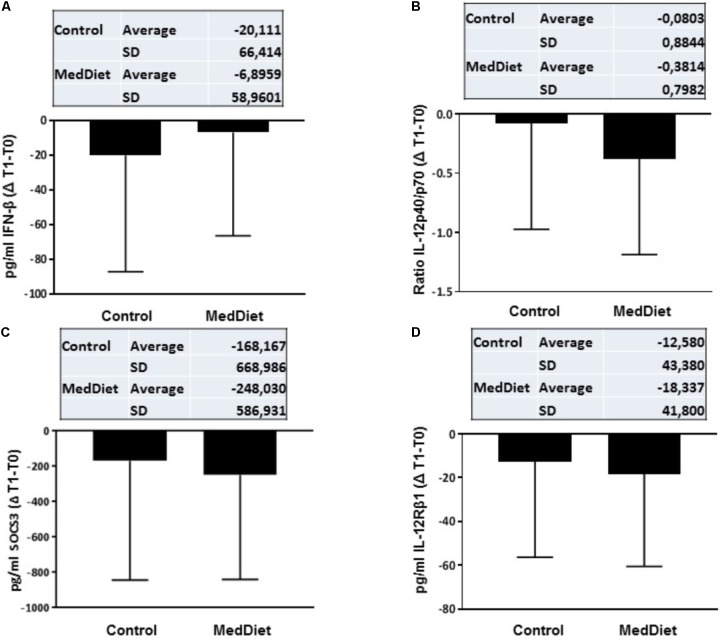
Cytokine expression following *in vitro* challenge with TLR agonists- Changes (Δ) of the parameter values in MedDiet and control diet at baseline and after 1-year of intervention. Δ of the parameters at baseline (T0) and after 1-year (T1) for IFN-β **(A)**, IL-12p40/70 ratio **(B)**, SOCS3 **(C)**, and IL-12Rβ1 **(D)** concentration were calculated and compared. For each parameter, the mean ± SD of the Δ at T0 and T1 for the control group and MedDiet group is shown in the relevant inset.

Following *in vitro* TLR stimulation, levels of selected cytokines were assessed in the culture supernatants. Overall, the MedDiet intervention did not significantly affected TLR-induced cytokine production (**Table 4**). On the other hand there were trends toward increased production of IFN-β (OR 1.98, 95%CI 0.86–4.53, *P* = 0.1056), and decreased production of IL-12Rβ1 (OR 0.50, 95%CI 0.22–1.13, *P* = 0.0929), SOCS3 (OR 0.50, 95%CI 0.21–1.18, *P* = 0.1108) and the ratio between IL-12p40/IL-12p70 (OR 0.54, 95%CI 0.26–1.16, *P* = 0.1122) (**Figures 3, 4**). Gender stratification did not reveal any differences in cytokine production. Taken together these data suggested that a prolonged period of exposure to the MedDiet might result in more profound changes in the immunological parameters evaluated.

**Table 4:** Effects of MedDiet on *in vitro* production of cytokines.

Cytokine	OR (95%Cl)	*P-*value
IFNα	1.49 (0.54–4.11)	0.4415
IFNβ	1.98 (0.86–4.53)	0.1056
IFNγ	1.19 (0.51–2.75)	0.6854
IL-12p40	0.84 (0.38–1.83)	0.6582
IL-12p70	1.62 (0.74–3.52)	0.2228
IL-12p40/IL-12p70	0.54 (0.26–1.16)	0.1122
IL-12Rβ1	0.50 (0.22–1.13)	0.0929
IL-12Rβ2	1.04 (0.40–2.66)	0.9396
SOCS3	0.50 (0.21–1.18)	0.1108


*Peripheral blood mononuclear cell were cultured with a cocktail of TLRs agonists (PolyA:U, Imiquimod, ODN-M362, LPS, Pam2CSK4) for 22 h and levels of cytokines determined in culture supernatants by ELISA assay. Odd ratio (OR) and 95%Cl and values are shown*

### T Cell Proliferation, CD8 T Cell Degranulation and Granulocyte Oxidative Burst Activity

First, the T cell proliferation response to 6-day SEB stimulation was compared to cells cultured in the absence of any stimulation. There were no significant differences between the control and MedDiet intervention groups, when comparing the change of T cell proliferation after 1 year of intervention (**Table 5**). Also, CD8 T cell degranulation was measured as % of CD107a expression on CD3^+^CD8^+^ cells induced by SEB stimulation, and the oxidative burst activity of granulocytes measured as % oxidizing cells within the granulocyte population; the MedDiet had no effect in altering both parameters after 1 year of intervention (**Table 5**), although a trend toward increased degranulation in the MedDiet intervention group was observed. No gender-specific differences were noted.

**Table 5:** Effects of MedDiet on T cell proliferation, CD8 T cell degranulation and granulocyte oxidative burst activity.

Phenotype	OR (95%Cl)	*P-*value
T Cell proliferation (G2 + M Phase)	0.67 (0.27–1.65)	0.3817
% CD8 T cell degranulation	2.13 (0.77–5.91)	0.1396
Oxidative burst test (GeoMean)	2.03 (0.53–7.70)	0.2922


*Peripheral blood mononuclear cell were treated in vitro as described in Section “Materials and Methods” using PBMC for T cell proliferation and CD8 degranulation or whole blood for granulocyte burst activity. Odd ratio (OR) and 95% confidence intervals (Cl) for % CD8 T cell degranulation, and percentage (%) of T cell proliferation (G2 + M phase) and Geomean of oxidative burst test. P-values are shown.*

### Dendritic Cell Subtypes

**FIGURE 5:**
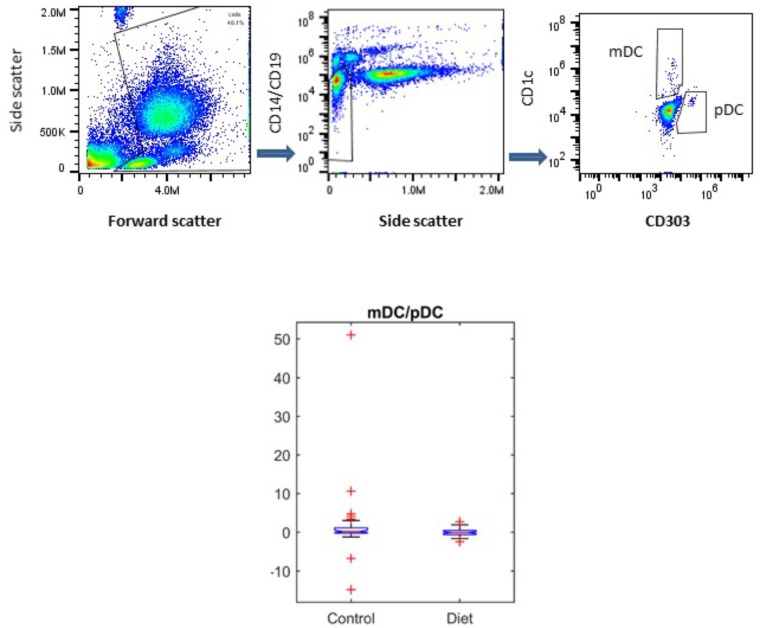
Effects of MedDiet on dendritic cell (DC) subpopulations. Whole blood was used at baseline and at the end of the 1-year dietary intervention to determine myeloid (m)DC and plasmacytoid (p)DC ratio. Representation of the gating strategy for the DCs enumeration is shown in **(A–C)**. **(D)** Boxplot showing the difference in the control and diet groups for mDC/pDC ratio right panel. A decrease, but not statistically significant of the ratio of mDC/pDC (*P* = 0.0536) was observed.

It has been shown that DC populations are affected by the aging process, and we have previously shown that the MedDiet had no effect on the numbers of mDCs or pDCs (Jing et al., 2009; Clements et al., 2017). Here we determined whether the MedDiet intervention had an impact on the ratio between mDCs and pDCs. DC subpopulations were identified and enumerated by using four-color flow cytometry in whole blood. **Figure 5** shows a trend toward decrease, but not statistically significant, in the ratio of mDC/pDC (*P* = 0.0536).

### Expression of Co-stimulatory Molecules on Freshly Isolated PBMC

**FIGURE 6:**
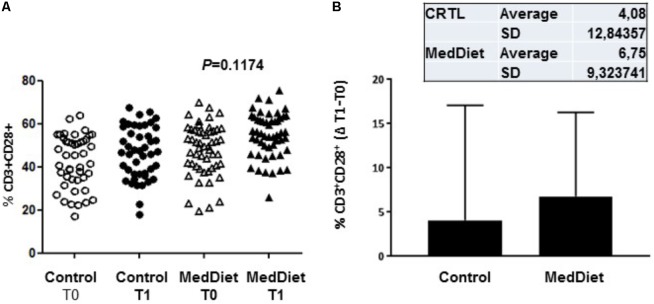
Expression of co-stimulatory molecules on freshly isolated PBMC. PBMCs were stained with CD3 and CD28 antibodies. All individual values for both the MedDiet and control group at T0 and T1 are shown **(A)**. A trend toward higher frequency of CD3^+^CD28^+^ cells in the MedDiet intervention group was seen in both genders (OR 2.19, 95%CI 0.92–5.21, *P* = 0.1174). Also, changes (Δ) of parameter values in MedDiet and control diet at baseline and after 1-year of intervention were determined (mean ± SD in inset) and compared **(B)**.

We then sought to investigate the effects of the MedDiet on the expression of the co-stimulatory molecules CD28, KIRDL4 (CD158d), CD161 and TCR Vα24-Jα18 (NKT) on freshly isolated PBMCs. We observed that 1-year intervention had no effect on the expression of these immunoregulatory molecules between the different study groups (**Table 6**). However, a trend toward higher frequency of CD3^+^CD28^+^ cells in the MedDiet intervention group was seen in both genders (OR 2.19, 95%CI 0.92–5.21, *P* = 0.1174) (**Figure 6**).

**Table 6:** Effects of MedDiet on co-stimulatory Molecules.

Phenotype	OR (95%Cl)	*P-*value
CD28	1.03 (0.47–2.26)	0.9387
KIR2DL4 (CD158)	1.14 (0.53–2.45)	0.7443
CD161	1.09 (0.26–4.59)	0.9116
TCRVα24-Jα18 (NKT)	0.91 (0.38–2.17)	0.8352


*Freshly collected PBMCs were stained for expression of costimulatory molecules by incubation with fluorochrome-labeled monoclonal antibodies. Odd ratio (OR) and 95% Cl and p-values are shown.*

### Plasma Biomarkers

Plasma was used to monitor the levels of the regulatory cytokines IL-9, IL-23, IL-2, IL-22 prior and after the 1-year dietary intervention. The cytokines IL-9 and IL-23 in plasma were above the lower limit of detection in only 4 of the 115 volunteers analyzed. IL-2 was present in more than 90% and IL-22 in more than 60% of the plasma samples analyzed. The statistical analysis of IL-2 and IL-22 did not show any significant MedDiet-associated change (**Table 7**).

**Table 7:** Effects of MedDiet on plasma levels of regulatory cytokines.

Cytokine	OR (95%CI)	*P*-value
IL-2	0.87 (0.40–1.86)	0.7133
IL-9	–	–
IL-22	0.66 (0.28–1.55)	0.3427
IL-23	–	–


Baseline and post-dietary intervention plasma levels of cytokines were determined using Luminex instrumentation. Odd ratio (OR) and 95% Cl and p-values are shown.

## Discussion

The MedDiet has been shown to down-regulate cellular and soluble inflammatory biomarkers related to the atherogenic process in subjects at high risk of CVD (Mena et al., 2009; Casas et al., 2014) and metabolic syndrome (Esposito et al., 2004; Fragopoulou et al., 2010). However, the immunological outcomes have not been extensively studied. It is important to highlight that the MedDiet was supplemented with Vitamin D_3_ (10 μg/day, 400 IU). The reason for giving a low dose vitamin D supplement was to minimize differences between study sites part of the whole NuAge project, which covered North, South, East and West Europe in which habitual diet and sunlight exposure affect vitamin D status. This combined intervention design does not allow defining the relative influence of the MedDiet and/or vitamin D on innate immune parameters. However, it has to be stressed that previous study showed that higher daily dose of vitamin D supplementation appeared to be effective on innate immunity and T cell reactivity (Prietl et al., 2013; Bashir et al., 2016). In our volunteers, adherence to a MedDiet diet for 1 year led to significant increase in the expression of CD40^+^CD86^+^ lymphocytes in women with a BMI < 31. The initiation of an adaptive immune response, requires multiple signals, including the expression of costimulatory molecules. CD40 is expressed in B cells and APCs and contribute to activate T cells through the CD40 ligand, in the absence of CD40 many cellular and immune functions are defective (Quezada et al., 2004). CD86 is also necessary for T cell activation and survival, is the ligand for CD28 and CTLA-4 and works in tandem with CD80. As these costimulatory molecules are important in immune activation, an association with gender may be related to the fact that women exhibit stronger cellular- and humoral-mediated immune responses compared to men, with a higher risk of autoimmune disease.

Within the MedDiet group we observed that the ratio of IL-12p40/IL-12p70 tended to decline. IL-12 up-regulates the expression of its own receptor, and elderly individuals have increased production of IL-12. The biological activity of the IL-12 is driven by the heterodimeric molecule IL-12p70, while IL-12p40 may antagonize IL-12. Indeed, total IL-12, IL-12p40, and the ratio IL-12p40/IL-12p70 increase significantly in aging (Rea et al., 2000). So, a decrease of the ratio IL-12p40/IL-12p70 may improve IL-12 functionality in elderly people. If this was the case, it is plausible that increased functionality of IL-12 might contribute to inflammageing. Also, a trend of decreased secretion of IL-12Rβ1 in the intervention group was observed. IL-12Rβ1 is a type 1 transmembrane receptor physically associated with the p40-domain common to both IL-12/IL23 and promotes their respective signaling pathways (Robinson, 2015). Regarding the trend in higher IFN-β but lower SOCS3 production in the MedDiet intervention group, IL-12 and IFN- β both signal through STAT4 phosphorylation, and a defective type I IFN response is described in T cells from elderly people (Li et al., 2015). Higher production of type I IFN may improve the immune response of elderly people against infection. Upregulation of TGF-β would therefore result in suppression of pro-inflammatory IL-12 and, also SOCS3 that down-regulates the STAT4-mediated IL-12 signaling by binding to the STAT4 docking site in IL-12Rβ2 (Yamamoto et al., 2003). SOCS3 proteins are constitutively expressed in naïve T helper cells and tends to decrease after cell stimulation. In aging, expression of SOCS3 is higher than in young people and does not decrease after stimulation (Tortorella et al., 2006). Our results showing a trend toward lower expression of SOCS3 after TLR stimulation would suggest the potential of MedDiet to exert beneficial effects.

DCs in human peripheral blood are divided in two subsets. Myeloid (m)DC are characterized by the production of high levels of IL-12 when stimulated with tumor necrosis factor-α or CD40 ligand and trigger a Th1-polarized immune response. Plasmacytoid (p)DCs drive an IL-4-independent Th2 polarization of naive T cells and secrete high levels of type I IFN, and IL-12 in response to certain bacteria (Stout-Delgado et al., 2008). In the elderly people, a decrease in pDCs but not mDCs (Shodell and Siegal, 2002; Pérez-Cabezas et al., 2007; Jing et al., 2009) was reported although others have reported conflicting results (Della Bella et al., 2007). However, it appeared that in frail individuals both populations are affected (Jing et al., 2009; Clements et al., 2017). Our data did not show any significant change in the mDCs and pDC populations in the control group compared to the intervention group, but it would appear that the mDC/pDC ratio tend to decrease. This finding suggests a T_H_2 predisposition of immune responses in the MedDiet group related to a polarized mDC:pDC balance toward pDCs, but a prolonged intervention study would be needed to confirm and possibly extend this observation.

The number, phenotype, and functions of NK cells are affected by the aging process in humans (Gayoso et al., 2011) and NKT cell numbers are increased (Peralbo et al., 2007). T cells from aging individuals showed increased expression of KIR whose expression is mostly found on senescent memory T cells that have lost CD28 expression (Li et al., 2008). Cells that produce IL-17 express CD161, and in the elderly, there is a decrease in expression of CD161+CD4 T cells (Lee et al., 2011). Our study shows no change in the expression of these markers following MedDiet intervention.

The majority of studies on aging to date have involved at-risk populations such as, for example individuals with pre-existing hypertension or metabolic syndrome. Instead our study focused on healthy elderly that did not have any signs of chronic disease or significantly impaired immune function; it is likely that this approach might have made more difficult to observe striking beneficial effects of the MedDiet.

## Conclusion

After 1 year of adherence to a Mediterranean-like dietary pattern with vitamin D_3_ supplements the only significant changes observed were in female participants with a BMI < 31. However, it is important to stress there was a tendency toward improvement of certain importance in some of the inflammaging-related parameters measured, such as a greater T cell response after TLRs stimulation. For the other parameters studied, it is likely that in the absence of overt risk factors or overly compromised immune function overt benefits cannot be detected after a relatively short-term adoption of the MedDiet.

## Ethics Statement

This study was approved by the National Research Ethics Committee East of England (12/EE/0109). All subjects gave written informed consent in accordance with the Declaration of Helsinki.

## Author Contributions

CN, KI, SRC, AS, SF-T, and CF designed the study. MM, KI, SJC, and JD performed the experimental work and data analysis. AJ and RG coordinated recruitment of volunteers and collected biological specimens. KI, CN, MG, and MM wrote the manuscript. CN supervised research. All authors participated to the finalization of the manuscript.

## Conflict of Interest Statement

The authors declare that the research was conducted in the absence of any commercial or financial relationships that could be construed as a potential conflict of interest.

## Footnotes

1 www.nu-age.eu

2 http://www.nu-age.eu/

3 https://www.gov.uk/government/publications/sacn-dietary-reference-values-for-energy
